# 

**Published:** 1894-10

**Authors:** 


ESTABLISHED 1855. SUBSCRIPTION, $2.00.

### ATLANTA MEDICAL AND SURGICAL JOURNAL.

OLD SERIES, VOL. XXVI. NEW SERIES, VOL. XI.

LUTHER B. GRANDY, M.D.
MANAGING EDITOR.

M. B. HUTCHINS, M.D.,
BUSINESS MANAGER.

ATLANTA, GA., OCTOBER, 1894. No. 8.

